# GRAde: a long-read sequencing approach to efficiently identifying the *CYP11B1*/*CYP11B2* chimeric form in patients with glucocorticoid-remediable aldosteronism

**DOI:** 10.1186/s12859-022-04561-w

**Published:** 2022-01-10

**Authors:** Yu-Ching Wu, Chia-I Chen, Peng-Ying Chen, Chun-Hung Kuo, Yi-Hsuan Hung, Kang-Yung Peng, Vin-Cent Wu, Jyy-Jih Tsai-Wu, Chia-Lang Hsu, TAIPAI group

**Affiliations:** 1 Department of Medical Research, National Taiwan University Hospital, Taipei, Taiwan; 2 Department of Internal Medicine, National Taiwan University Hospital, Taipei, Taiwan; 3 Graduate Institute of Oncology, National Taiwan University College of Medicine, Taipei, Taiwan; 4 Graduate Institute of Medical Genomics and Proteomics, National Taiwan University College of Medicine, Taipei, Taiwan; 5 TAIPAI, Taiwan Primary Aldosteronism Investigator Group and TSA, Taiwan Society of Aldosteronism, Taipei, Taiwan

**Keywords:** Glucocorticoid-remediable aldosteronism, Chimeric genes, Long-read sequencing

## Abstract

**Background:**

Glucocorticoid-remediable aldosteronism (GRA) is a form of heritable hypertension caused by a chimeric fusion resulting from unequal crossing over between 11β‐hydroxylase (*CYP11B1*) and aldosterone synthase (*CYP11B2*), which are two genes with similar sequences. Different crossover patterns of the *CYP11B1* and *CYP11B2* chimeric genes may be associated with a variety of clinical presentations. It is therefore necessary to develop an efficient approach for identifying the differences between the hybrid genes of a patient with GRA.

**Results:**

We developed a long-read analysis pipeline named GRAde (GRA deciphering), which utilizes the nonidentical bases in the *CYP11B1* and *CYP11B2* genomic sequences to identify and visualize the chimeric form. We sequenced the polymerase chain reaction (PCR) products of the *CYP11B1*/*CYP11B2* chimeric gene from 36 patients with GRA using the Nanopore MinION device and analyzed the sequences using GRAde. Crossover events were identified for 30 out of the 36 samples. The crossover sites appeared in the region exhibiting high sequence similarity between *CYP11B1* and *CYP11B2*, and 53.3% of the cases were identified as having a gene conversion in intron 2. More importantly, there were six cases for whom the PCR products indicated a chimeric gene, but the GRAde results revealed no crossover pattern. The crossover regions were further verified by Sanger sequencing analysis.

**Conclusions:**

PCR-based target enrichment followed by long-read sequencing is an efficient and precise approach to dissecting complex genomic regions, such as those involved in GRA mutations, which could be directly applied to clinical diagnosis. The scripts of GRAde are available at https://github.com/hsu-binfo/GRAde.

**Supplementary Information:**

The online version contains supplementary material available at 10.1186/s12859-022-04561-w.

## Background

Primary aldosteronism (PA) is the most common and curable form of secondary arterial hypertension. Most diagnosed cases of PA are mainly caused by either aldosterone overproduction by both adrenal glands or unilateral aldosterone-producing adenomas (APA) [1]. However, about 5% of cases are inherited forms of familial hyperaldosteronism (FH) [2]. According to current understanding, there are three well-established forms of FH (FH-I–III), and some germline mutations associated with PA, such as CACN1H and CACN1D, have been identified [3]. However, data from genetic analyses reveal a more complex situation, and more heritable forms of PA may still be undiscovered [2].

**Fig. 1:**
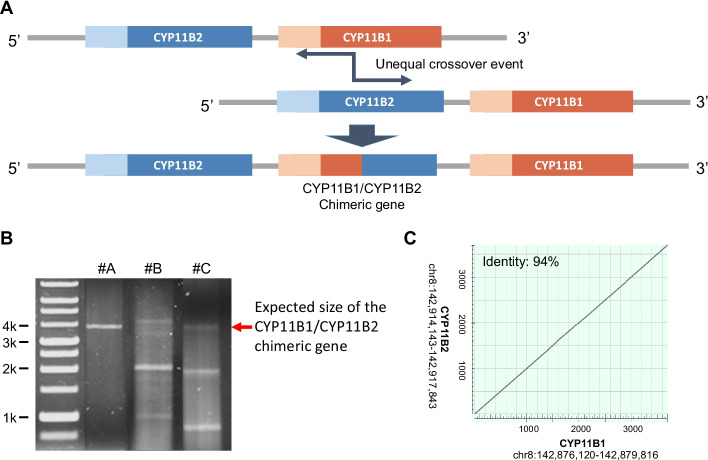
The challenge of *CYP11B1*/*CYP11B2* chimeric form identification. **A** Scheme of the *CYP11B1*/*CYP11B2* chimeric gene. **B** Long-range PCR shows the PCR products of the *CYP11B1*/*CYP11B2* chimeric gene and unexpected fragments. Amplification of a chimeric gene is expected to produce a 3.9 kb product. Case A has a clear single band of the expected size of the PCR product, but cases **B** and **C** have multiple bands with weak signals of the expected size of the main product. **C** Dot plot revealing the high degree of similarity between the *CYP11B1* and *CYP11B2* genomic sequences. The plot was generated by blast2seq using default parameters

Familial hyperaldosteronism type I (FH1), also called glucocorticoid-remediable aldosteronism (GRA), is transmitted as an autosomal-dominant disorder and accounts for 0.5–1.0% of PA cases [4]. GRA is caused by a chimeric gene resulting from a nonhomologous crossing-over event on chromosome 8q24.3, between the 11β‐hydroxylase (*CYP11B1*) and aldosterone synthase (*CYP11B2*) genes. This chimeric enzyme contains the promoter of *CYP11B1* at the 5′ end and the coding sequences from *CYP11B2* at the 3′ end. It can therefore synthesize aldosterone under adrenocorticotropic hormone (ACTH) control (Fig. 1A).

Although GRA is a genetic disease, the clinical and biochemical characteristics of patients are highly variable, and even patients from the same familial type may present different symptoms [5, 6]. In addition, different crossover patterns of the chimeric *CYP11B1*/*CYP11B2* gene within a familial type have been described [7]. These findings suggest that variability in clinical presentations might be related to heterogeneity in the hereditary factor—in particular, in the crossover pattern of the hybrid gene. Currently, the genetic diagnosis of GRA is made using Southern blotting or long-range polymerase chain reaction (PCR) techniques [8–12]. However, these methods are unable to precisely determine the crossover pattern. Therefore, it is crucial to develop a more precise method for identifying the specific type of hybrid gene that is carried by a patient.

The detection of pathogenic gene fusion in inherited diseases and oncology is particularly useful. In general, fusion events cause a loss or gain of function in one of the fused partners. However, unlike with oncogenic fusion genes, it is relatively difficult to detect gene conversion in genes with highly similar sequences. New detection strategies for this kind of gene fusion are urgently required to facilitate diagnostic and therapeutic decisions.

Since the introduction of next-generation sequencing (NGS), tremendous progress has been achieved in all fields of biology. The declining costs and growing availability of NGS have made it the method of choice for genetic analysis and related applications. Nevertheless, NGS suffers from a limited availability of data on several aspects, such as repetitive elements, polymorphic regions, camouflaged genes, and large structural variations (SVs), which prevents full extraction of information associated with the genome [13, 14]. However, recently developed single-molecule sequencing techniques such as single-molecule real-time sequencing (SMRT) and nanopore sequencing provide access to larger variations, because the read lengths are typically several thousands of bases [15, 16]. These techniques provide the opportunity to investigate or diagnose diseases caused by pathogenic structural variations and gene conversion, such as GRA. In this study, we used Oxford nanopore technology (ONT) to sequence the PCR products of chimeric *CYP11B1*/*CYP11B2* genes and developed a long-read analysis pipeline that can efficiently identify and visualize the chimeric forms.

## Results

### The challenge of GRA chimeric form identification

Previous studies have suggested that a variety of *CYP11B1*/*CYP11B2* chimeric forms are associated with different clinical presentations [5, 6]. Long-range PCR is one of the standard approaches to GRA diagnosis. However, in a few of GRA cases, multiple bands with weak signals for the expected PCR products are obtained (Fig. 1B), making diagnosis difficult. To avoid misdiagnosis of patients with GRA, it is crucial to identify the exact crossover site. The current method for identifying crossover sites is multiplex PCR with Sanger sequencing [12]. However, the genomic sequences of *CYP11B1* and *CYP11B2* are about 94% identical in the main crossover region, making primer design for the enrichment of regions that are specific to only one of the two genes challenging (Fig. 1C). In addition, this procedure is time-consuming and laborious. To address this issue, we proposed an alternative strategy that combines long-range PCR with nanopore sequencing to confirm the chimeric gene and identify the crossover site, and we developed an analysis pipeline to decipher these long-read sequences.

### Overview of GRAde

**Fig. 2:**
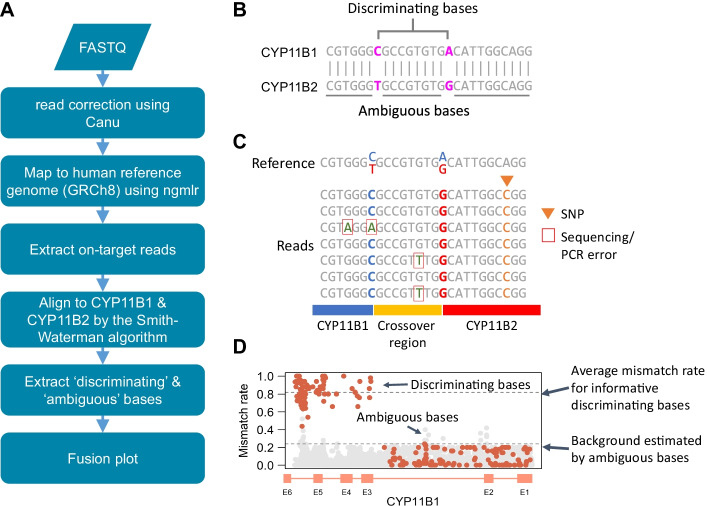
Overview of GRAde. **A** Analysis workflow of GRAde. **B** The identification of discriminating and ambiguous bases in the *CYP11B1* and *CYP11B2* genomic sequences. **C** The concept of using discriminating and ambiguous bases to recognize the *CYP11B1* and *CYP11B2* genes. **D** An example of a fusion plot based on the *CYP11B1* genomic sequence. Red and grey points represent the discriminating and ambiguous bases

The analysis workflow of GRAde is illustrated in Fig. 2A. The input for GRAde was FASTQ files, which were generated from the long sequencing reads of PCR products derived from GRA samples. This pipeline consists of two components: quality control for the sequencing reads, and the procedure for determining the crossover regions of the *CYP11B1*/*CYP11B2* chimeric form. High-quality reads were obtained by correcting them using a nonhybrid approach, Canu [17], and then mapping them to the human reference genome. Only the reads that aligned with the loci of *CYP11B1* and *CYP11B2* were considered for further analysis. To accurately dissect each qualifying read, GRAde used the Smith–Waterman algorithm to align reads to the *CYP11B1* and *CYP11B2* genomic sequences, respectively, and then analyzed the alignment results to identify the crossover region.

Due to the high degree of sequence similarity between the *CYP11B1* and *CYP11B2* genes, we first identified the “discriminating” and “ambiguous” bases by comparing them to the *CYP11B1* (chr8:142876120–142879816) and *CYP11B2* (chr8:142914143–142917843) sequences (Fig. 2B). We then used the discriminating bases to distinguish the sequences from the genomic source (Fig. 2C). There were 180 and 188 discriminating bases for *CYP11B1* and *CYP11B2*, respectively (Additional file 1). The median of the positional distribution of the neighbor discriminating bases was 11 bp, with a range of 1–221 bp. Ideally, the ambiguous bases should perfectly match both genes, but there are typically some mismatches due to errors in the PCR or sequencing processes and genetic polymorphisms (Fig. 2C). Mismatches of ambiguous bases may affect the interpretation, so the ambiguous bases were discarded if polymorphisms were reported at these positions in the single-nucleotide polymorphism (SNP) database (dbSNP). Based on the alignment results of all reads mapped to *CYP11B1* and *CYP11B2*, we calculated the mismatch rates of each discriminating and ambiguous base and visualized them as a fusion plot (Fig. 2D). Because of the relatively high error rate of nanopore sequencing, if the bases aligned to discriminating sites did not exactly match either *CYP11B1* or *CYP11B2*, we considered them to be sequencing or PCR errors and did not include them in the fusion plot. This fusion plot provides the intuitive and interpretable chimeric form for each sample.

### Variety of *CYP11B1*/*CYP11B2* chimeric forms in GRA samples

**Fig. 3:**
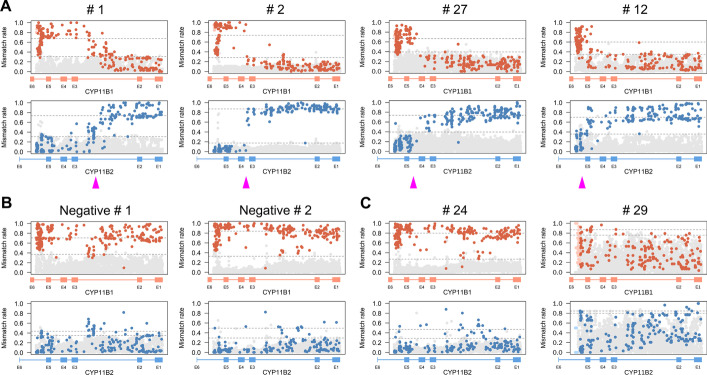
A variety of cross-over regions could be identified using GRAde. Fusion plots for **A** representative fusion forms, **B** negative controls (normal *CYP11B2* gene), and **C** false-positive cases that were diagnosed with GRA based on PCR but without any fusion observable in the sequences. The pink triangle denotes the possible cross-over region

We collected 36 samples from patients who were diagnosed with GRA based on clinical practice guidelines, which was confirmed by using the long-range PCR technique to reveal the chimeric genes. The chimeric genes were amplified using the long-range PCR technique and then subjected to nanopore sequencing. We also amplified *CYP11B2* genes from six other patients with PA as negative controls. The results of the GRAde analysis of the 36 GRA samples are summarized in Table 1, and the fusion forms are shown in Fig. 3A and Additional file 2. Sixteen of the 36 cases had fusion sites located at intron 2, and the crossover region of the other cases was within the ranges of exon 3–intron 3 (seven cases), exon 4–intron 4 (five cases) and exon 5–intron 5 (two cases). There were no fusion patterns in the fusion plots for any of the negative control samples of normal *CYP11B2* genes (Fig. 3B). Notably, there were also six GRA cases for whom no fusion patterns were apparent (Fig. 3C and Additional file 2), suggesting that their diagnoses might be based on false-positive test results.

**Table 1:** GRAde analysis results for GRA samples

Sample no.	Crossover region	Sample no.	Crossover region
#1	E2-I2	#19	E2-I2
#2	E3-I3	#20	E2-I2
#3	E2-I2	#21	E4-I4
#4	E3-I3	#22	No fusion observed
#5	E2-I2	#23	No fusion observed
#6	E3-I3	#24	No fusion observed
#7	E2-I2	#25	E2-I2
#8	E3-I3	#26	E3-I3
#9	E3-I3	#27	E4-I4
#10	E3-I3	#28	E2-I2
#11	E2-I2	#29	No fusion observed
#12	E5-I5	#30	E2-I2
#13	No fusion observed	#31	E5-I5
#14	No fusion observed	#32	E4-I4
#15	E2-I2	#33	E2-I2
#16	E2-I2	#34	E2-I2
#17	E2-I2	#35	E2-I2
#18	E4-I4	#36	E4-I4

E2-I2: located in exon 2 and intron 2

E3-I3: located in exon 3 and intron 3

E4-I4: located in exon 4 and intron 4

E5-I5: located in exon 5 and intron 5

No fusion observed: no clear fusion pattern was observed in the GRAde fusion plot

### Validating the crossover site via multiplex PCR with Sanger sequencing

**Fig. 4:**
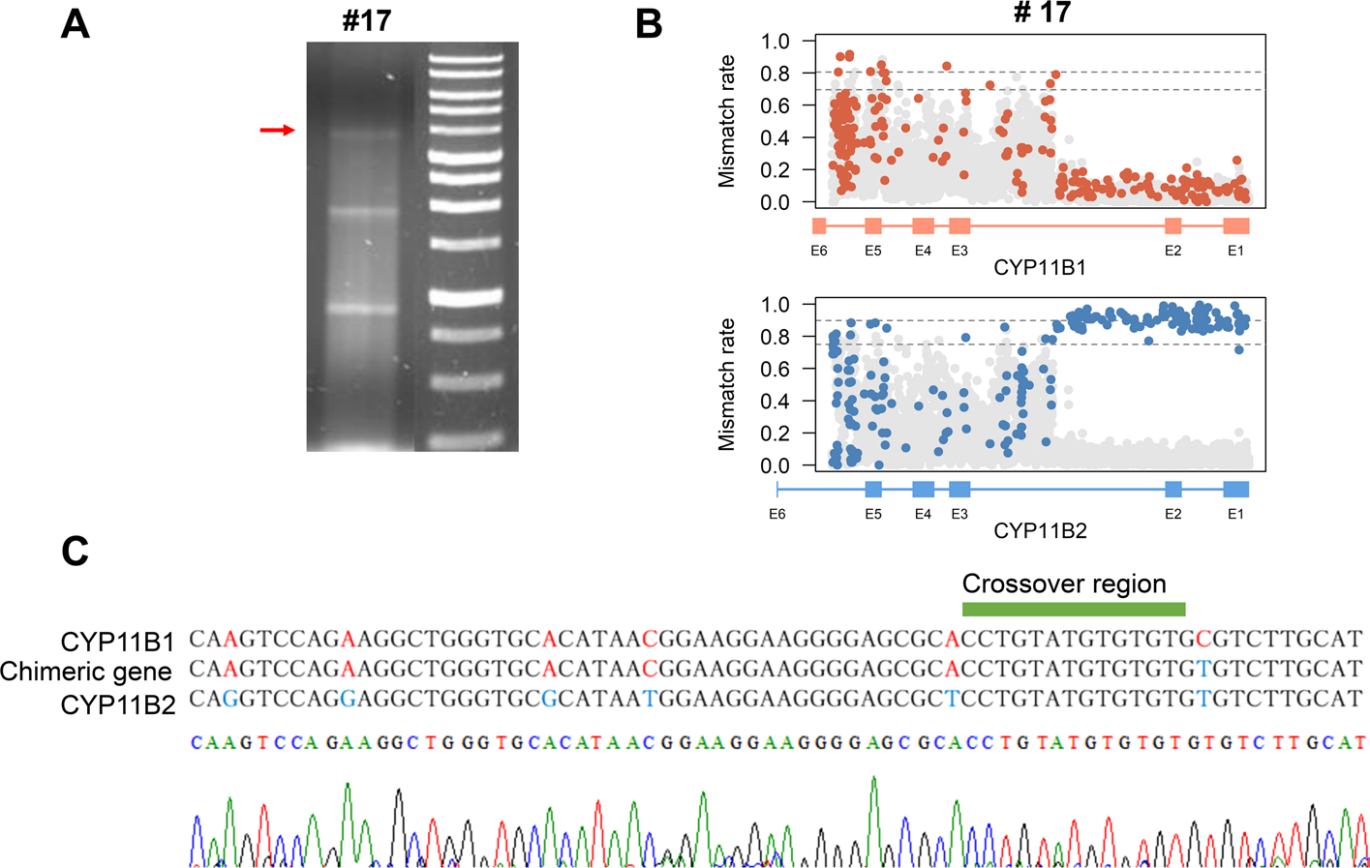
A case in which the cross-over region was validated by Sanger sequence analysis. **A** Long-range PCR revealed PCR products of the *CYP11B1*/*CYP11B2* chimeric gene in sample #17. Amplification with a chimeric gene is expected to produce a 3.9 kb product. **B** The fusion plot of sample #17 reveals a possible cross-over region located in intron 2, with high background noise. **C** Sequencing of the chimeric PCR product demonstrated that the crossover site was located in intron 2. The nucleotides that differed between *CYP11B1*, *CYP11B2*, and the chimeric gene are highlighted in different colors

To demonstrate the validity of our approach and GRAde, we selected a case for whom the PCR produced multiple products and the signal for the chimeric gene was relatively weak (Fig. 4A). After sequencing the PCR products using the nanopore technology and analyzing the reads using GRAde, a clear fusion pattern was revealed in the fusion plot, although the background noise was high at the 3′ end of the chimeric gene (Fig. 4B). Using the fusion plot, we designed primers to amplify the identified crossover region and sequenced the amplified products via Sanger sequencing. Nucleotide sequence analysis showed that the gene-conversion site was in the middle of intron 2, which was consistent with the GRAde result.

### Runtime and robustness of GRAde

We evaluated the runtime and robustness of GRAde for use as a diagnostic tool. We generated testing sets that were randomly sampled from case No. 34, with a varying number of reads (100, 200, 500, 1000, 2000, or 3000). As shown in Additional file 3, analysis of input with 3000 reads could be completed within six minutes, and the most time-consuming step was performing the Canu algorithm for hybridization-based sequence correction. We were able to achieve stable results using the sample with 200 reads, so we consider that to be the minimum number of reads required for analysis (Additional file 3).

## Discussion

The emerging long-read sequencing technologies offer improvements in the characterization of genetic variation and regions that are difficult to assess using short reads. These techniques have been used to investigate genetic disorders with previously known or strongly suspected disease loci [18] and are considered a diagnostic tool. The general strategy of long-read–based diagnosis is to enrich the locus of causality and then perform long-read sequencing. Long-range PCR is a technique currently used for the detection of GRA [8–12], so we chose it as the enrichment method. However, GRA is a special case because the locus of causality is formed by two genes with a high degree of sequence similarity (Fig. 1C). This results in challenging primer design and poor PCR products for evaluation, due to issues like the presence of multiple bands and weak signals for the expected chimeric products (Fig. 1B). Also, the presence of the expected band in the PCR product does not necessarily mean it is a chimeric gene, which can lead to incorrect diagnoses based on false-positive test results (Fig. 3C). Therefore, long-range PCR in addition to long-read sequencing is a more efficient method of chimeric gene detection than traditional Sanger sequencing and may reduce the false-positive rate of traditional PCR testing.

However, PCR-based enrichment still has several limitations. PCR amplification can be influenced by improper PCR conditions [19, 20], thus producing false negatives or incorporating PCR errors. Analysis of sequencing reads from PCR products with multiple bands may also be hampered by a high level of background noise and an unclear fusion pattern (Fig. 3A). To avoid this, the development of alternative target-enrichment methods for GRA chimeric genes is required, such as capture-based [21, 22] and CRISPR-based enrichment methods [23, 24].

In our GRA cohort, there were six patients who had PCR products for sequencing in which we could identify no fusion pattern. The possible reasons for this include poor integrity and low purity of the genomic DNA, which could have resulted from the state of storage and the process of DNA extraction. Other possible factors include poor specificity of the primers, inappropriate DNA input, and nucleic-acid contamination. It is difficult to design extremely specific primers for the crossover region of chimeric genes, so this pipeline can assist in excluding nonspecific PCR products and distinguishing the correct signal.

In addition to the *CYP11B1*/*CYP11B2* chimeric gene, there are some SNPs at the *CYP11B1* and *CYP11B2* loci that are also associated with hypertension [25]. Theoretically, these single nucleotide variants (SNV) can be detected in the current long-read sequence data. To provide precise diagnosis and treatment of GRA, however, it is critical to report all possible variants in genetic testing. Although GRAde was developed for the identification of chimeric forms, therefore, we will incorporate the function of variant-calling to identify SNVs and small insertions and deletions (indels).

Although GRAde is specifically designed for the identification of crossover sites between *CYP11B1* and *CYP11B2* in GRA patients, the analysis strategy used in GRAde could also be applied to other diseases that are caused by dysfunctional proteins due to unequal crossover events. For example, chronic granulomatous disease (CGD) is caused by the chimeric form of *NCF1* (neutrophil cytosolic factor 1) and its pseudogenes *NCF1B* (neutrophil cytosolic factor 1B pseudogene) and *NCF1C* (neutrophil cytosolic factor 1C pseudogene) [26]. Because these two pseudogenes are on either side of *NCF1* and have 99% sequence identity to *NCF1*, distinguishing *NCF1* from its pseudogenes in CGD patients relies on a set of SNPs [27] and an analysis similar in concept to that of GRAde. In addition to CGD, several other diseases are caused by the chimeric products of the crossover between a gene and its pseudogenes, such as congenital adrenal hyperplasia, caused by the chimeric genes *CYP21A1P*/*CYP21A2* [28], and Gaucher disease, caused by a fusion gene formed from *GBA* (glucosylceramidase beta) and its pseudogene *GBAP1* (glucosylceramidase beta pseudogene 1) [29]. GRAde could easily be modified and used for crossover-site identification in other diseases. Besides the detection of specific fusion genes, our strategy could be also applied to genome-wide detection of this kind of gene fusion by systematically identifying discriminating bases in homologous genes.

## Conclusions

In this study, we proposed the strategy of combining long-range PCR with long-read sequencing techniques to identify gene conversions, such as the one that causes GRA. This approach is not only more efficient than general multiplex PCR followed by Sanger sequencing, but also reduces the false-positive rate for PCR-based genetic testing. This analysis procedure could be applied to the diagnosis of other diseases caused by unequal crossover between two genes with highly similar sequences.

## Methods

### Patients

This study was approved by the institutional review board of the National Taiwan University Hospital, Taipei, Taiwan (No. 200611031R) (ClinicalTrials.gov number NCT00746070). All participants provided written informed consent before inclusion in the study. The Taiwan Primary Aldosteronism Investigation (TAIPAI) group enrolled possible PA patients who first had their aldosterone-to-renin ratio (ARR) screened for PA detection and were then followed-up. Screening, confirmation, and subtype identification of the PA were performed in hypertensive patients according to the standard TAIPAI protocol and aldosteronism consensus [30–33]. Fulfillment of the following three conditions confirmed a diagnosis of PA: (1) autonomous excess aldosterone production evidenced by an ARR > 35; (2) a TAIPAI score [34] of > 60%; (3) seated post-saline loading plasma aldosterone concentration (PAC) > 16 ng/dL [35], or PAC/plasma renin activity (PRA) > 35 (ng/dL)/(ng/mL/h) in a post-captopril/losartan test [30].

### Sample preparation

For the detection of the chimeric gene, PCR was performed using the method described by MacConnachie et al. [12] with some modifications. We used the following primer sets to amplify the normal *CYP11B2* gene and the chimeric *CYP11B1*/*CYP11B2* gene with PfuUltra II Fusion HS DNA Polymerase (Agilent): forward: 5′CAGGTCCAGAGCCAGTTCTCCCAT/reverse: 5′ACCCTCCTTCTCCTTGTACACCCA forward: 5′CAGTTCTCCCATGACGTGATCCCT/reverse: 5′ACCCTCCTTCTCCTTGTACACCCA.

The touchdown PCR process was as follows: 95 °C for 2 min; 38 cycles of denaturation at 95 °C for 1 min; annealing at 70–61 °C for 1 min; and extension at 72 °C for 5 min and 72 °C for 3 min*.* The annealing temperature began at 70 °C and was lowered by 1 °C every two cycles until it reached 61 °C; this annealing temperature was maintained until the end of the cycling process. The PCR amplicons were evaluated in a 0.8% agarose gel, cleaned up with the 0.45X Agencourt AMPure XP beads (Beckman Coulter), and quantified using a Qubit fluorometer (Life Technologies).

### Sample barcoding

For simultaneous detection of multiple samples, we used the ligation method to tag the amplified sample with the native barcoding adaptor (Oxford Nanopore Technologies), which allows up to 24 different libraries (barcodes 1–24) to be combined and loaded onto a single flow cell at the same time. The amplified fragment end was repaired and dA-tailed using the End Repair/dA-tailing Module (KAPA Roche). The end-repaired product was purified using 1X Agencourt AMPure XP beads. Next, a unique dT-tailed barcode adaptor was ligated on the dA-tailed template using ligation Master Mix (KAPA Roche). The barcoded samples were then purified with 0.45X Agencourt AMPure XP beads. The quality and quantity of each sample were evaluated using Nanodrop and a Qubit fluorometer, respectively. The barcoded samples were equally pooled for the sequencing library preparation.

### Library preparation and sequencing

For the construction of the sequencing library, we used the KAPA hyper prep kit (Roche). First, the amplified and barcoded samples were pooled together, and end repair and dA-tailing were performed using the End Repair/dA-tailing Module. The end-repaired product was purified using 1X Agencourt AMPure XP beads. Next, adapter ligation and tethering were carried out with sequencing adapter (Oxford Nanopore Technologies) and ligation Master Mix. The sequencing-adapter–ligated DNA library was then purified with 0.5X Agencourt AMPure XP beads, Adapter Bead binding buffer (Oxford Nanopore Technologies) was added, and the samples were eluted in the Elution Buffer (Oxford Nanopore Technologies).

Before sequencing, the sequencing-adapter–ligated DNA library was mixed with Library Loading beads, and SpotON Flow Cells (R9.4) (FLO-MIN106D) were primed with running buffer. The samples were run on a MinION sequencing device for approximately 24 h, and the sequencing runs were operated by the MinKNOW software. Base-calling from the electrical data generated by the sequencer was performed using Guppy (v3.0.3) and the resulting raw sequence data were demultiplexed using qcat (v1.1.0).

### Implementation of GRAde

Reads with lengths of 3000–5000 bp were considered for downstream analysis. Reads were corrected using a nonhybrid-based approach Canu (v1.4) [17] with the default parameters altered only as follows: genomeSize = 5 k, overlapper = mhap, utgReAlign = true, and stopOnReadQuality = false. Corrected reads were compared to the human reference genome (GRCh38) using ngmlr (v0.2.7) using default parameters [36]. The reads that aligned on the loci of *CYP11B1* and *CYP11B2* were defined as on-target reads. Each read was aligned to the conserved regions of *CYP11B1* and *CYP11B2* using the Smith–Waterman algorithm, implemented by the SSW library [37]. The discriminating and ambiguous bases were extracted from the alignment between *CYP11B1* and *CYP11B2* by the Smith–Waterman algorithm. The mismatch rate of each discriminating and ambiguous base was calculated by parsing the alignment results of reads aligned to *CYP11B1* and *CYP11B2*. The background noise was defined as the 99th percentile of the mismatch rate of all ambiguous bases, which is represented by the lower dashed line in the fusion plot. The foreground, represented by the upper dashed line in the fusion plot, was the average of the mismatch rates of the discriminating bases that had mismatch rates higher than the background. In addition, the mismatch rates of the discriminating bases were fitted using a sigmoid function to identify the crossover site, using the drm function implemented in the drc R package. All scripts were implemented in Python (v2.7) and R (v3.5.1).

## Supplementary Information

**Additional file 1:** Nucleotides that differed between *CYP11B1* and *CYP11B2*.

**Additional file 2:** Fusion plots for all GRA patients.

**Additional file 3:** Runtime and robustness of GRAde.

## Data Availability

The scripts used for the analyses have been deposited at GitHub and are available at https://github.com/hsu-binfo/GRAde.

## Abbreviations

ACTH: Adrenocorticotropic hormone
APA: Aldosterone-producing adenomas
ARR: Aldosterone-to-renin ratio
CGD: Chronic granulomatous disease
CRISPR: Clustered regularly interspaced short palindromic repeat
FH: Familial hyperaldosteronism
GRA: Glucocorticoid-remediable aldosteronism
Indel: Insertion and deletion
NGS: Next-generation sequencing
ONT: Oxford Nanopore Technology
PA: Primary aldosteronism
PAC: Plasma aldosterone concentration
PCR: Polymerase chain reaction
PRA: Plasma renin activity
SMRT: Single-molecule real-time sequencing
SNP: Single-nucleotide polymorphism
SNV: Single nucleotide variant
SV: Structural variation
TAIPAI: The Taiwan Primary Aldosteronism Investigation
