# Supplementary figures and images for: GRAde: a long-read sequencing approach to efficiently identifying the CYP11B1/CYP11B2 chimeric form in patients with glucocorticoid-remediable aldosteronism
Source: BMC Bioinformatics. 2022 Jan 10;22(Suppl 10):613. doi: 10.1186/s12859-022-04561-w (PMC8750845; doi:10.1186/s12859-022-04561-w)

Fusion site at E2-I2

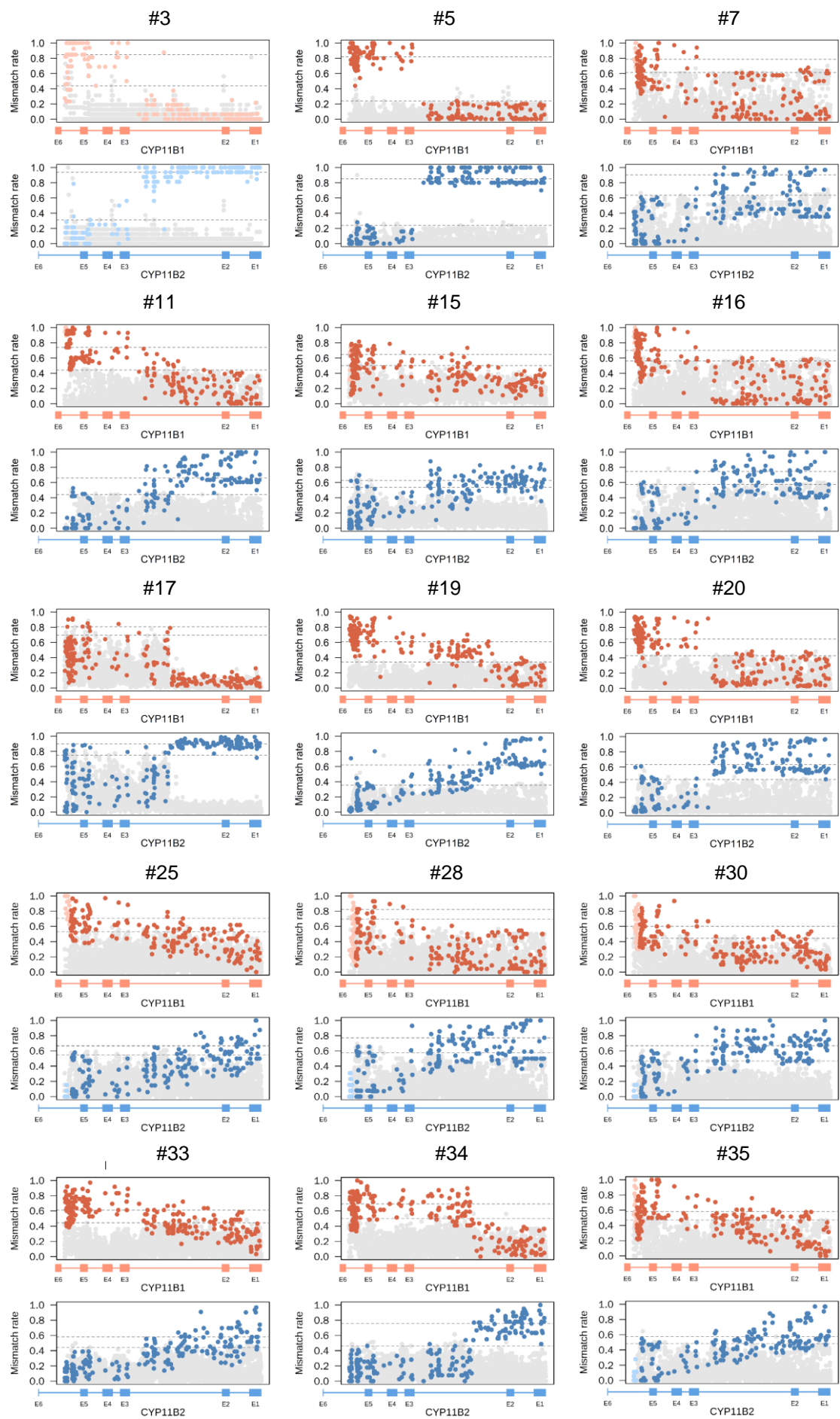

Fusion site at E3-I3

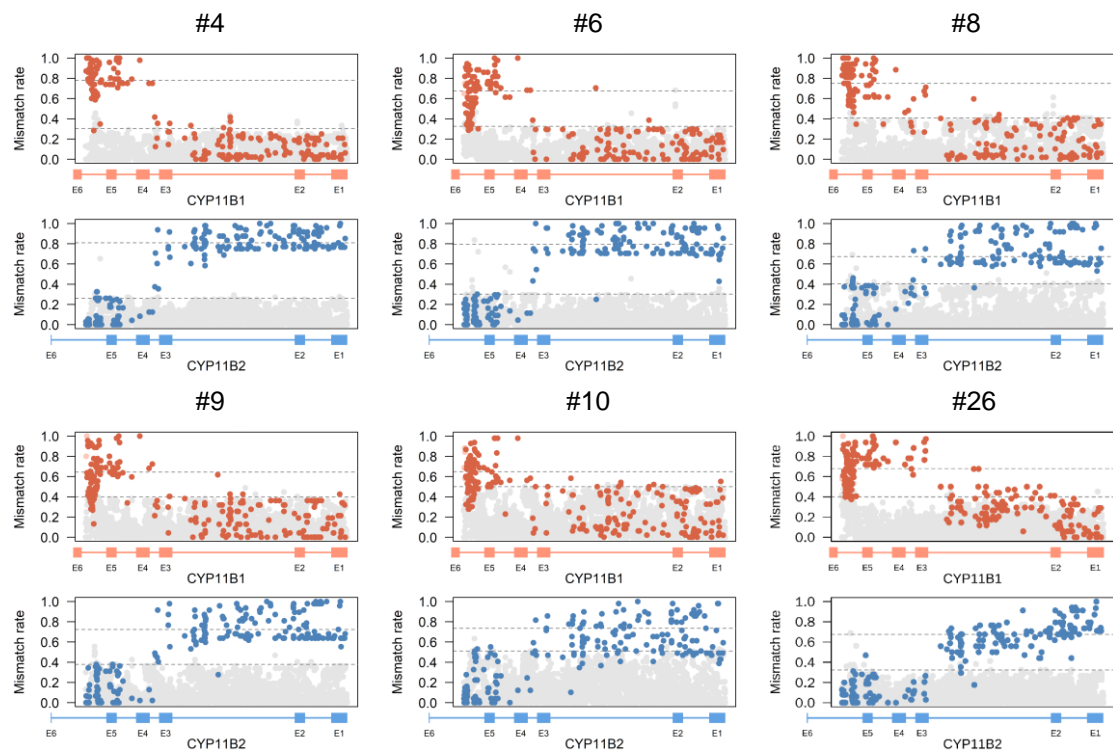

Fusion site at E4-I4

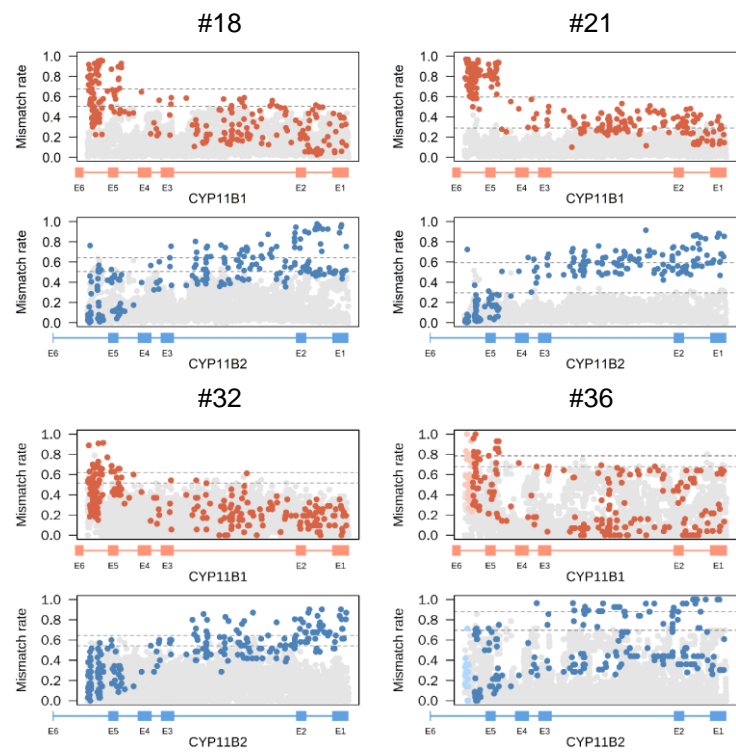

Fusion site at E5-I5

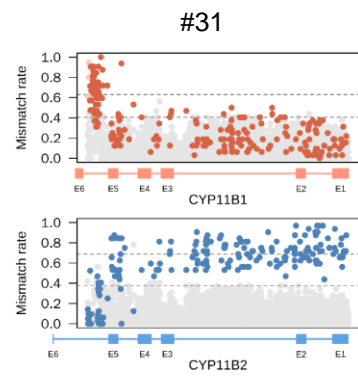

No fusion pattern

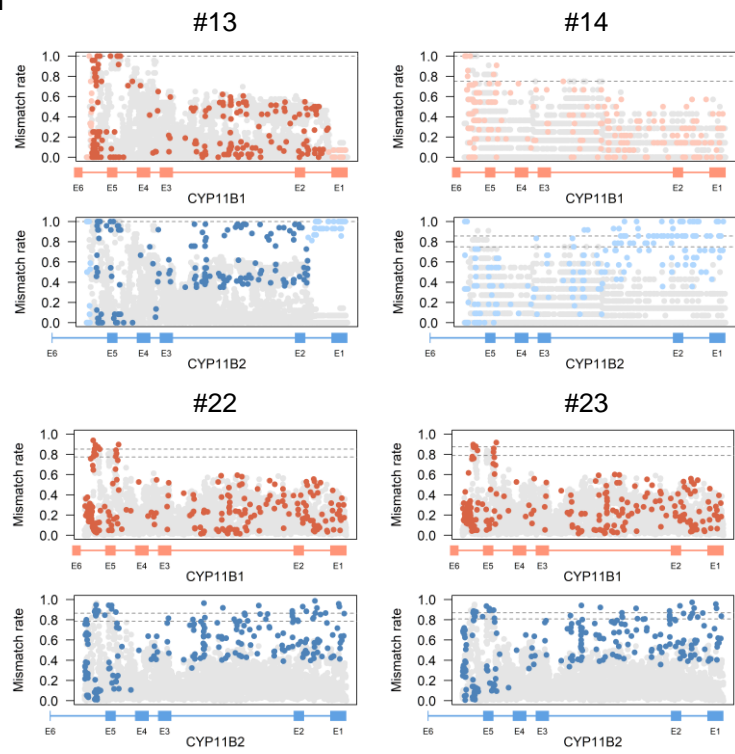

Supplement: Supplementary file 2 — Additional file 2: Fusion plots for all GRA patients. [file 12859_2022_4561_MOESM2_ESM.pdf]
