# Supplementary material for: GRAde: a long-read sequencing approach to efficiently identifying the CYP11B1/CYP11B2 chimeric form in patients with glucocorticoid-remediable aldosteronism
Source: BMC Bioinformatics. 2022 Jan 10;22(Suppl 10):613. doi: 10.1186/s12859-022-04561-w (PMC8750845; doi:10.1186/s12859-022-04561-w)

Additional file 3 - **A**. The runtime of whole analysis pipeline (left), Canu part (middle), and Smith-Waterman alignment part (right) with different number of reads as input (n = 10). **B**. the evaluation of robustness.

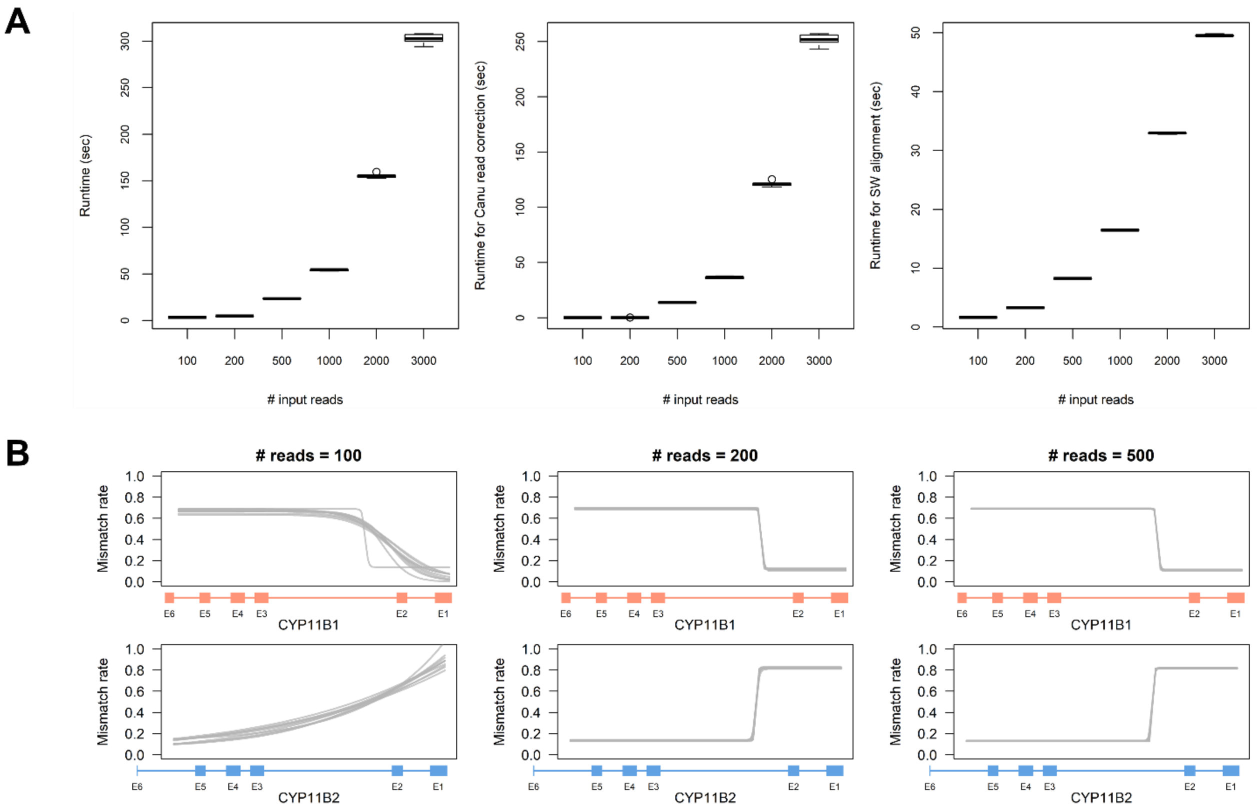

Supplement: Supplementary file 3 — Additional file 3: Runtime and robustness of GRAde. [file 12859_2022_4561_MOESM3_ESM.pdf]
